# Prognostic value of DNA aneuploidy in gastric cancer: a meta-analysis of 3449 cases

**DOI:** 10.1186/s12885-019-5869-9

**Published:** 2019-07-02

**Authors:** Jing Xu, Ruolin Zhu, Lulu Fan, Shangqing Ge, Wei Wei, Xiaoqiu Li, Liangshan Da, Zhenya Jia, Zhiyan Zhao, Jie Ning, Jie Da, Wanren Peng, Kangsheng Gu, Guoping Sun

**Affiliations:** 1 Department of Medical Oncology, The First Affiliated Hospital of Anhui Medical University, 218 Jixi Road, Hefei, 230000 Anhui Province China; 2 The Department of Neurology, The First Affiliated Hospital of Anhui Medical University, 218 Jixi Road, Hefei, 230000 Anhui Province China

**Keywords:** Aneuploidy, Gastric cancer, Survival

## Abstract

**Background:**

DNA aneuploidy has attracted growing interest in clinical practice. Nevertheless, its prognostic value in gastric cancer patients remains controversial. This meta-analysis aims to explore the impact of DNA ploidy status on the survival of gastric cancer patients.

**Methods:**

We used PubMed and Web of Science databases to retrieve relevant articles. The correlation between DNA aneuploidy and the clinicopathological features of gastric cancer, such as stage, depth of invasion (T), lymph node metastasis (N), distant metastasis (M), differentiation (G), tumor types (Lauren classification) and overall survival (OS) were evaluated. Hazard ratios (HRs) with corresponding 95% confidence intervals (CIs) were collected carefully from each article OS was presented with HRs. The relationships between DNA aneuploidy and each characteristic were analyzed using risk ratios (RR) and a 95% confidence interval (CI). Significance was established using *P* < 0.05. Funnel plot was conducted to detect the publication bias.

**Results:**

After careful selection, 25 studies involving 3449 cases were eligible for further analyses. Patients with DNA aneuploidy were considered at risk of more advanced stages (stage III-IV vs. stages I-II, RR = 1.23; 95% CI, 1.07 to 1.42; *P* = 0.003), lymph node metastasis (N+ vs. N-: RR = 1.43; 95% CI, 1.12 to 1.82, *P* = 0.004), and intestinal tumor type (intestinal vs. diffuse: RR = 1.45; 95% CI, 1.02 to 2.06; *P* = 0.04). And an adverse relation was observed between DNA aneuploidy and tumor differentiation. While no association was found between DNA aneuploidy and distant metastasis (*P* = 0.42) nor depth of tumor invasion (*P* = 0.86). Regarding overall survival, aneuploid tumors were associated with worse survival in all patients (*P* < 0.00001).

**Conclusions:**

We found that DNA aneuploidy was an important predictor for gastric cancer patients, and should be used as a potential biomarker for further classification in gastric cancer.

**Electronic supplementary material:**

The online version of this article (10.1186/s12885-019-5869-9) contains supplementary material, which is available to authorized users.

## Background

Gastric cancer was the third leading cause of cancer-related death, and was responsible for 723,000 deaths all over the world [1]. At present, proliferation markers such as the histopathological TNM (tumor-node-metastasis) classification and Lauren classification have been extensively adopted for predicting survival in gastric cancer [2]. Nonetheless, there is a large variation in survival of gastric cancer patients with similar TNM stage or Lauren classification. Therefore, it is required to explore more precise markers at a molecular level to increase the precision of prognosis prediction for individual patients [3]. For decades there has been a growing interest in chromosomal instability, which was defined by DNA aneuploidy, to classify gastric carcinoma into molecular subtype groups [1].

DNA aneuploidy was found in the majority (70–90%) of cancer cells, and was defined as the set of an abnormal number of chromosomes in cells [4, 5]. DNA aneuploidy reflected a high genotypic instability, leading to malignant behavior of cells. Numerous articles which investigated DNA aneuploidy in various cancers have been published. Its role in non-small cell lung cancer, colorectal cancer and breast cancer seems clear. It was observed that patients with DNA aneuploid tumor had worse clinical outcomes in non-small cell lung cancer, colorectal cancer and breast cancer [6–9].

Previous studies [10–34] which focused on the correlation between DNA aneuploidy and survival in gastric cancer reported conflicting results. Consequently, we conducted this meta-analysis to explore the prognostic value of DNA aneuploidy status in gastric cancer patients.

## Methods

### Search strategy and study selection

Our meta-analysis was conducted following the Preferred Reported Items for Systematic Reviews and Meta- Analysis (PRISMA) statement [35]. We searched relevant articles from two databases (PubMed, and Web of Science March 20th 2018). Articles were identified using the following keywords: ‘DNA ploidy/aneuploidy’, ‘gastric cancer/carcinoma’ and ‘survival’. There was no restriction of English language during our search.

Articles were picked out on the basis of inclusion criteria as follows: 1. sufficient information of baseline clinicopathological characteristics of gastric cancer patients; 2. the research focused on the association between overall survival (OS) and DNA aneuploidy; 3. proven histological diagnosis of gastric cancer prior to anticancer therapies; 4. DNA aneuploidy status were presented specificly; 5. value of Hazard ratio (HR) with a 95% confidence interval (CI). Alternatively, the Kaplan-Meier (K-M) curve or a Cox regression model was provided..

Literature retrieval and selection was cautiously performed by two authors (JX and RZ). To avoid overlap of patient cohort, we included the latest studies with largest samples from the same organization. All authors reached consensus and made final decision through thorough discussion.

### Data extraction

Two authors (JX, RZ) independently extracted the useful data from all eligible articles. The collected information was listed as follows: the last name of the first author, publication date, sample size, mean or median age of population, DNA ploidy methods, specimens, disease stage, follow-up period, treatments and OS. The risk ratios (RRs) of DNA aneuploidy to tumor stage, depth of invasion (T), lymph node metastasis (N), distant metastasis (M), differentiation (G), tumor types (intestinal and diffuse, based on Lauren classification), and HRs of OS with 95% CIs were checked. If the value of HRs were not provided straightforwardly, survival data were estimated using K-M curves by Engauge Digitizer 4.1.

### Quality assessment of primary studies

The Newcastle-Ottawa Scale (NOS) scale was occupied to assess the quality of included papers.

### Statistical analysis

We conducted statistical analyses on the basis of the Cochrane Collaboration Guidelines. The pooled RRs were estimated by the Mantel-Haenszel’s method, whereas the pooled HRs were calculated by the inverse variance method. The homogeneity assumption was investigated by Cochran’s Q statistic. The Higgins’ *I*^2^ index was calculated to evaluate inconsistency among the included studies. If the heterogeneity was absent, the pooled RR and HRs were calculated by the fixed-effect model (*χ*^*2*^*P* < 0.100 and *I*^*2*^ < 50%). Otherwise the meta-analysis was performed by employing the DerSimonian random-effects model [36, 37]. Significance was identified if the value of *P* was lower than 0.05. Egger’s tests were generated to detect potential publication bias (*P >* 0.05 was considered representative of no statistically significant publication bias) [38]. All the statistical calculations of meta-analyses were performed by RevMan 5.3. And Egger’s tests were carried out through Stata11.0. All the *P* values were two-sided.

## Results

### Literature search

**Fig. 1:**
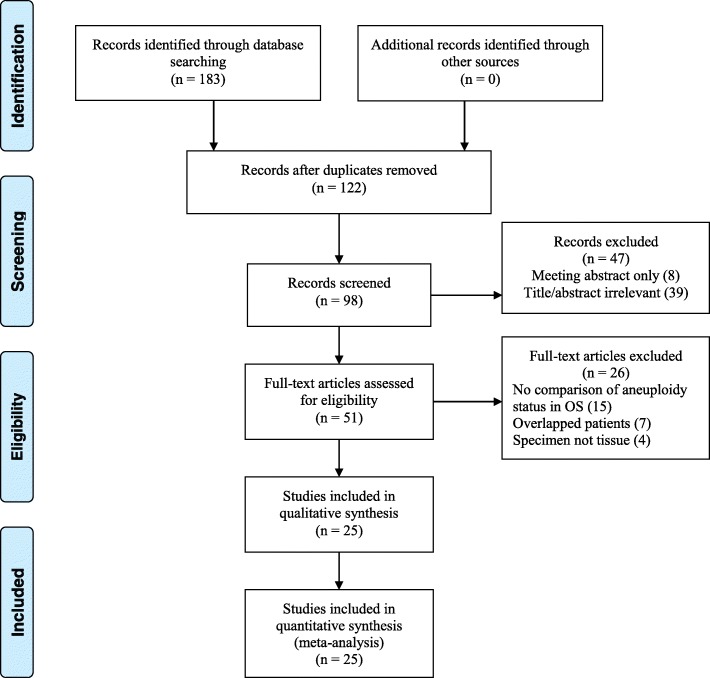
PRISMA flow diagram. OS, overall survival

According to the search strategy, 98 studies were identified for full text review, in which 25 studies [10–34] possessed sufficient data for following meta-analyses (Fig. 1). The main features of each study were shown in Table 1.

**Table 1:** Characteristics of included studies

Publications	No. of patients	Age (year)	Method	Specimen	Stage	Follow-up (month)	Treatment	neclei	Multiple sample form one case	NOS
Ballantyne 1987 [10]	77	67 (43–88)^a^	FCM	PES	NA	36	Surgery	> 20,000	NA	7
Sasaki 1989 [11]	70	58.1 (33–80)^b^	FCM	FTS	NA	48	Surgery	10,000	NA	7
Wyatt 1989 [12]	76	66 (40–88)^a^	FCM	PES	NA	60	Surgery	NA	NA	8
Baretton 1991 [13]	125	66.8 ± 11.3^b^	FCM	PES	NA	108	Surgery	NA	NA	8
Filipe 1991 [14]	116	64 (31–87)^a^	FCM	PES	NA	108	Surgery	> 10,000	NA	8
Kimura 1991 [15]	270	NA	FCM	PES	I-IV	60	Surgery	> 10,000	NA	8
Kakeji 1993 [16]	93	NA	NA	NA	I-IV	60	Surgery	100	No	8
Suh 1993 [17]	103	55.9 (29–70)^b^	FCM	PES	I-IV	24	Surgery	> 10,000	NA	7
Flyger 1995 [18]	97	67 (23–85)^a^	FCM	PES	NA	60	Surgery	10,000	NA	8
Tsuchiya 1995 [19]	127	NA	FCM	PES	NA	96	Surgery+CT chemotherapy	NA	No	8
Victorzon 1996 [21]	242	NA	NA	NA	I-IV	120	Surgery	NA	NA	8
Sakusabe 1996 [20]	216	56.7 (24–86)^b^	FCM	PES	III	60	Surgery	10,000	No	8
Imada 1997 [22]	88	62.4^b^	FCM	FTS	NA	36	Surgery+CT chemotherapy	> 10,000	Yes	7
Omejc 1997 [23]	76	NA	FCM	PES	I-IIIB	47 (14–67)^a^	Surgery	NA	Yes	8
Abad 1998 [24]	76	70^b^	FCM	PES	I-IV	43^a^	NA	10,000	Yes	7
Hirose 1998 [25]	142	59^b^	Cytofluorometry	PES	I-IV	132	Surgery	NA	NA	8
Danesi 2000 [26]	137	66.3^b^	FCM	PES	I-III	80.8 (42.9–111.5)^a^	Surgery	20,000	NA	8
Ikeguchi 2000 [27]	97	58 ± 13^b^	FCM	PES	II	60	Surgery	10,000	NA	8
Russo 2001 [28]	69	NA	FCM	PES	I-IV	95^b^	Surgery	NA	Yes	8
Wu 2005 [29]	60	59.6^a^	FCM	NA	NA	60	Surgery	NA	No	8
Nesi 2007 [30]	115	65.6 ± 11.3^b^	FCM	PES	I-IV	84	Surgery	> 20,000	Yes	8
Wiksten 2008 [31]	337	NA	FCM	PES	NA	150 (52.8–249.6)^a^	Surgery	> 10,000	No	8
Belien 2009 [32]	221	71 (34–96)^a^	FCM	PES	I-IV	21^a^	Surgery	NA	No	7
Syrios 2012 [33]	212	61^a^	ICM	PES	IV	65	Surgery+CT	200–300	NA	8
Nishimura 2017 [34]	207	A: 64.8 ± 10.9^b^ D: 62.1 ± 13.5^b^	LSC	PES	I-IV	60	RA	NA	NA	8

*A* aneuploidy, *D* diploidy, *PES* paraffin embedded specimen, *FTS* fresh tissue specimen, *FCM* flow cytometry, *ICM* image cytometry, *SCM* static cytometry, *LSC* laser scanning cytometry, *NA* not available; ^a^: median; ^b^: mean; CT: chemotherapy

3449 gastric patients diagnosed between 1968 and 2006 were evaluated for our meta-analyses. The population of patients in each study ranged from 60 to 337, with the follow-up period varying from 21 months to 20 years. Additional file 1: Table S1 displayed the specific characteristics of each meta-analysis. The definitions of DNA aneuploidy in these studies shared some common points as follows: DNA index (DI) reflected the ratio of DNA content in G0/1 cells to the reference G0/1 diploid peak. If a cell population displayed only one G0/G1 peaks (DI = 1), tumor was considered diploidy. Otherwise tumor with additional G0/G1 peaks was aneuploidy.

### DNA aneuploidy and tumor stage

**Fig. 2:**
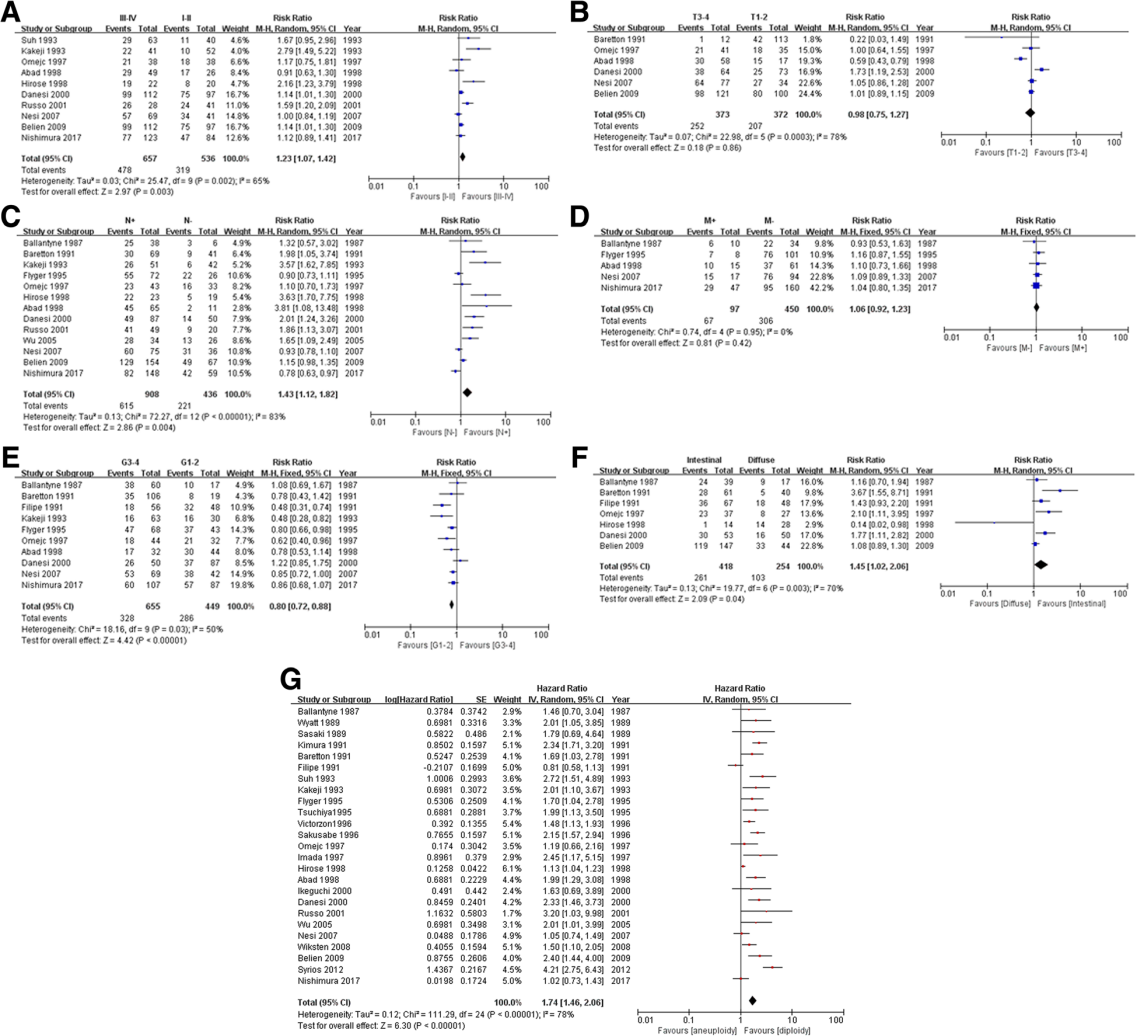
**a** Forest Plot of DNA aneuploidy rates in stage III-IV tumor vs. I-II tumor. **b** Forest Plot of DNA aneuploidy rates in T3-4 tumor vs. T1-2 tumor. **c** Forest Plot of DNA aneuploidy rates in tumor with lymph node metastasis vs. tumor without lymph node metastasis. **d** Forest Plot of DNA aneuploidy rates in tumor with distant metastasis vs. tumor without distant metastasis. **e** Forest Plot of DNA aneuploidy rates in G3-4 tumor vs. G1-2 tumor. **f** Forest Plot of DNA aneuploidy rates in intestinal tumor vs. diffuse tumor. **g** Forest Plot of DNA aneuploidy for overall survival

There were ten studies examining the association between DNA aneuploidy rate and tumor pTNM stages. It could be seen from Fig. 2a that DNA aneuploidy was more frequent in patients with stage III-IV gastric cancer than those with stages I-II tumors (RR = 1.23; 95% CI, 1.07 to 1.42; *P* = 0.003).

### DNA aneuploidy and depth of tumor invasion

Six studies reported the relationship between DNA aneuploidy and the depth of invasion. However, no association was found between DNA aneuploidy and depth of gastric cancer invasion (T3–4 vs. T1–2: RR = 0.98; 95% CI, 0.75 to 1.27; *P* = 0.86; Fig. 2b).

### DNA aneuploidy and lymph node metastasis

The correlation between DNA aneuploidy and lymph node metastasis was mentioned in thirteen studies. The pooled RR indicated that aneuploidy was more frequently observed in lymph node positive tumor than in lymph node negative ones (RR = 1.43; 95% CI, 1.12 to 1.82, *P* = 0.004; Fig. 2c).

### DNA aneuploidy and distant metastasis

Nevertheless, there was no evident relation between aneuploidy and distant metastasis (*P* = 0.42; Fig. 2d) from five relevant studies. The RR of patients with distant metastatic tumor compared to those without distant metastatic cancer was 1.06 (Fig. 2d).

### DNA aneuploidy and tumor differentiation

A total of ten studies with 1104 patients investigated data concerning the association between DNA aneuploidy status and tumor differentiation. The meta-analysis implied that DNA aneuploidy was remarkably more frequent in G1–2 tumors than in G3–4 tumors (G3–4 vs. G1–2: RR = 0.8; 95% CI, 0.72 to 0.88; *P* < 0.00001; Fig. 2e).

### DNA aneuploidy and tumor type

There were seven articles which studied the DNA aneuploidy frequency in intestinal compared with diffuse gastric cancer. The pooled analysis from these articles suggested a higher presence rate of DNA aneuploidy in intestinal tumors than in diffuse tumors (RR = 1.45; 95% CI, 1.02 to 2.06; *P =* 0.04; Fig. 2f).

### Meta-analysis for overall survival

For the entire gastric cancer population, the OS of patients with DNA aneuploid tumors was worse than that of patient with DNA diploid ones (25 studies; HR = 1.74; 95% CI, 1.46 to 2.06; *P* < 0.00001; Fig. 2g).

### Meta-analysis for different measurements of DNA aneuploidy

**Fig. 3:**
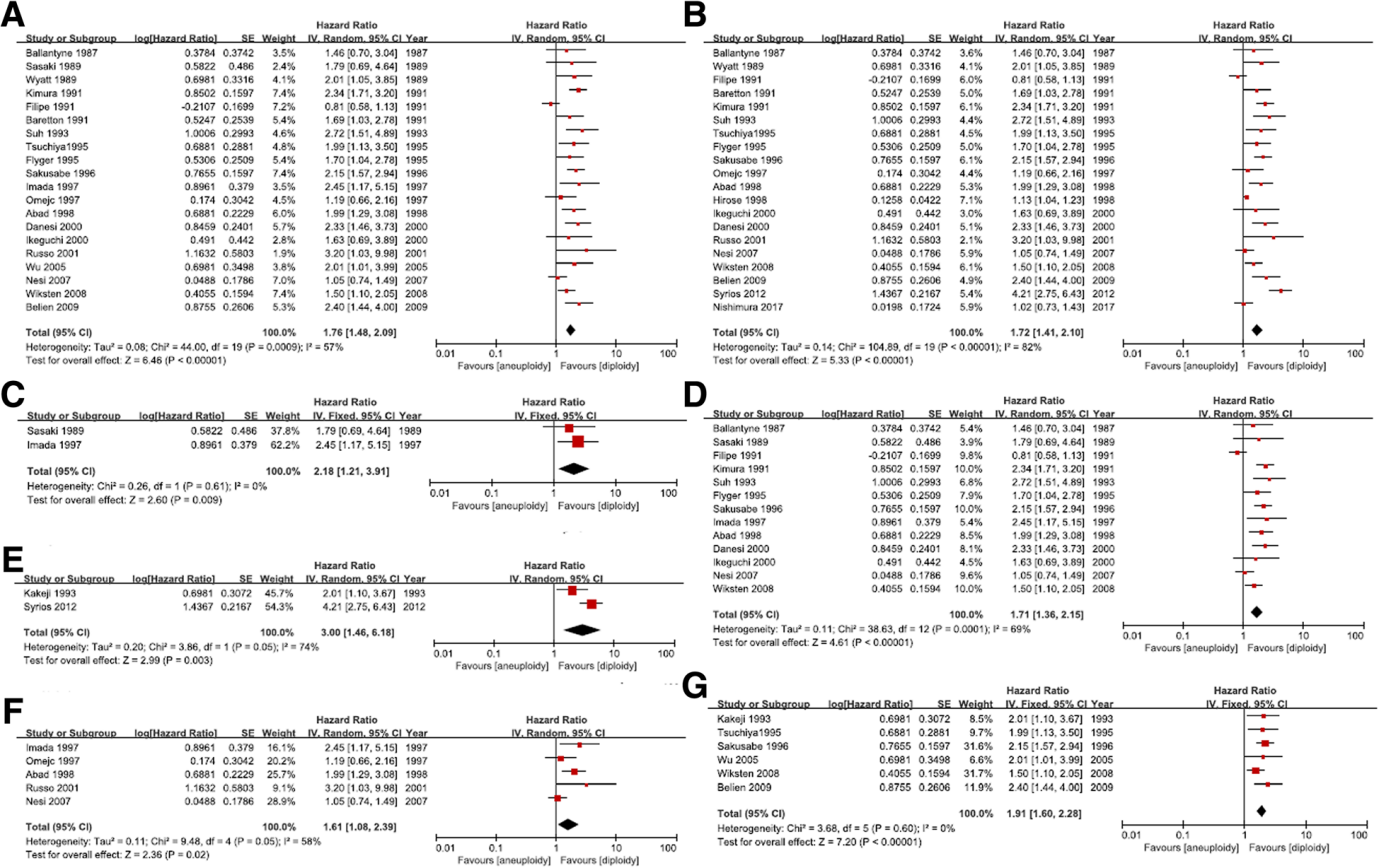
**a** Forest Plot of DNA aneuploidy for OS using flow cytometry. **b** Forest Plot of DNA aneuploidy for OS using paraffin embedded tissue. **c** Forest Plot of DNA aneuploidy for OS using fresh tissue specimen. **d** Forest Plot of DNA aneuploidy for OS: cell number>10000. **e** Forest Plot of DNA aneuploidy for OS: cell number<1000. **f** Forest Plot of DNA aneuploidy for OS: multiple samples per tumor. **g** Forest Plot of DNA aneuploidy for OS: single sample per tumor

Twenty studies utilized flow cytometry for DNA aneuploidy detection. The pooled analysis based on them exhibited a pronounced relation between DNA aneuploidy and gastric cancer survival (HR = 1.76; 95%CI, 1.48 to 2.09; *P* < 0.00001; Fig. 3a).

A total of 20 studies used paraffin embedded tissues (PFS), while only 2 studies used fresh tissues (FTS). DNA aneuploidy status in both specimens showed significant prognostic value for gastric cancer patients (PFS: *P* < 0.00001, Fig. 3b; FTS: *P* = 0.009, Fig. 3c).

We analyzed survival data not only for studies with large scale of cell numbers (nuclei more than 10,000, Fig. 3d), but also for studies with small scale of cell numbers (nuclei less than 1000, Fig. 3e). The *P* values for both groups were less than 0.01.

Tumors contain massive heterogeneity including of aneuploidy. Multiple samples per tumor were supposed to be tested in order to give an overall picture of the genomic stability. Detailed information was listed in Table 1. Five studies used multiple samples (HR = 1.61; 95%CI, 1.08 to 2.39; *P* = 0.02; Fig. 3f), six studies used single sample (HR = 1.91; 95%CI, 1.60 to 2.28; *P* < 0.00001; Fig. 3g), while others gave no specific information. No matter what kind of sample was used, DNA aneuploidy was still significantly associated with worse survival.

### Publication bias

Funnel plots were used to detect the publication bias which did not reveal remarkable asymmetry. In addition, there was also no considerable publication bias detected by Egger’s tests. The *P*-values for Egger’s test of each category were listed in Additional file 1: Table S1.

## Discussion

The analysis of DNA ploidy is based on the evaluation of cell viability, which exhibits the percent of DNA in S phase cells. Nevertheless, various factors, e.g., the overlap between diploid cells and aneuploid cells, background fragments, and insufficient number of cells, might disturb the evaluation of S phase. Therefore, the assessment of DNA aneuploidy is usually applied for the analysis of DNA content. Although DNA aneuploidy was supposed to play a crucial role in varies cancers, its prognostic value in gastric cancer was still under debate. To the best of our knowledge, our study is the first meta-analysis exploring the prognostic value of aneuploidy in gastric cancer patients.

Recently, antitumor strategy mainly relies on the TNM system and histologic classification which are common prognostic factors for the disease-free survival and overall survival in cancer patients. However, patients sharing the same clinicopathologic characteristics may present various clinical outcomes. It has been suggested that the measurement of DNA nuclear content via flow cytometry may help distinguish gastric cancer patients who have different disease relapse risk and cancer-related death risk [39–41]. Nevertheless, the results of previous studies were often influenced by the heterogeneity in the composition and size of the samples. From our study, the prognostic value of DNA aneuploidy in GC is clearer.

DNA aneuploid tumors were usually regarded as high metastatic risk tumors with aggressive behaviors [42, 43]. We did not, however, find any associations between depth of tumor invasion and distant metastases. Likewise, there is a negative association between aneuploidy and tumor differentiation. It may suggest that the malignant potential of a cell clone does not directly rely on the DNA amount. Moreover, only one sample was extracted from each tumor patient, which may lead to a relatively low incidence of DNA aneuploidy.

Our study revealed that DNA aneuploidy was remarkably correlated with advanced TNM staging and lymph-node metastases. This further indicated that DNA aneuploidy played a critical role in tumor progression and aggressiveness. This also suggested the combination of these three factors were crucial to predict the clinical course of gastric cancer patients.

Diffuse tumor was supposed to have a poorer prognosis compared with intestinal tumor [44]. Interestingly our results show a remarkable correlation between DNA aneuploidy and intestinal histotype rather than the diffuse type. Such heterogeneity may suggest that in these two different tumor types, DNA aneuploidy have various biological and clinical prognostic implications. According to Wyatt et al.’s study, intestinal tumor patients with DNA aneuploidy suffered from shorter survival [12]. Further studies of this patient population are required to validate this association.

There were some advantages of our meta-analyses. For example, our data processing was based on sufficient literature retrieval and eligible statistical methods. Moreover, we adopted NOS scale to evaluate all the included articles, and found that the quality of their data was relatively high. Nevertheless, some limitations in our study should be mentioned. Firstly, our analyses were based on published studies instead of individual data. Secondly, the included studies were not randomized work. Some of them were prospective studies, while the rest were retrospective studies. Thirdly, the enrolled patients were diagnosed from 1968 to 2006. In this period, the treatment for gastric cancer improved greatly. Fourthly, the methodological change in aneuploidy measurements across these years is a confounding factor. Fifthly, the methodological differences in each study might affect the final conclusion. Subgroup analyses on DNA aneuploidy measurements, cell numbers and specimen preparations were performed. We found that these factors did not influence the association between DNA aneuploidy and clinical outcomes of gastric cancer. Sixthly, stomach contains acid and enzymes which might affect the sample preparations for DNA aneuploidy detection. This factor could affect the obtained data in each study. To avoid this, some of the studies specified the time limit to fix the tissues. And others used deep site or multiple sites of tumor for preparation. However, a standard is required in the future experiments. Our meta-analysis provide an indication of the prognostic value of DNA aneuploidy, we hope our study could encourage future studies to draw a standard guideline in this aspect.

## Conclusion

In summary, here we reported that DNA aneuploidy might serve as an important biomarker for gastric cancer. DNA aneuploidy could provide detailed prognostic information for different tumor type and stage. Although further experiments will be required to provide more evidence, our findings will be helpful for personal treatment strategy in gastric cancer patients.

## Additional file

Additional file 1: **Table S1.** Outcomes of each category. (DOCX 17 kb)

## Data Availability

Not applicable. Our study is a systematic meta-analysis all of the data were related to the studies listed in in the Table 1.

## Abbreviations

CI: Confidence interval
G: Differentiation
HR: Hazard ratio
K-M: Kaplan-Meier
M: Distant metastasis
N: Lymph node metastasis
NOS: Newcastle-Ottawa Scale
OS: Overall survival
PRISMA: Preferred Reported Items for Systematic Reviews and Meta- Analysis
RR: Risk ratios
T: Depth of invasion
TNM: Tumor-node-metastasis
