# Supplementary material for: Prognostic value of DNA aneuploidy in gastric cancer: a meta-analysis of 3449 cases
Source: BMC Cancer. 2019 Jul 2;19:650. doi: 10.1186/s12885-019-5869-9 (PMC6607593; doi:10.1186/s12885-019-5869-9)
Supplement: Supplementary file 1 — Table S1. Outcomes of each category. (DOCX 17 kb) [file 12885_2019_5869_MOESM1_ESM.docx]

**Table S1** Outcomes of each category

| **Outcome** | **No. of studies** | **No. of patients** | **Effect estimate (95% CI)** | ***P* for Egger’s test** |
| --- | --- | --- | --- | --- |
| Stage III-IV vs. I-II | 10 | 1121 | 1.23(1.07,1.42) | 0.313 |
| T3-4 vs.T1-2 | 6 | 745 | 0.98(0.75,1.27) | 0.216 |
| N+ vs. N- | 13 | 1344 | 1.43(1.12,1.82) | 0.033 |
| M+ vs.M- | 5 | 547 | 1.06(0.92,1.23) | 0.427 |
| G3-4 vs.G1-2 | 10 | 1104 | 0.80(0.72,0.88) | 0.206 |
| Intestinal vs. Diffuse | 7 | 672 | 1.45(1.02,2.06) | 0.850 |
| Overall survival | 25 | 3449 | 1.74(1.46,2.06) | 0.500 |

T：depth of invasion; N: lymph node metastasis; M: distant metastasis; G: differentiation.
